# Supplementary material for: GRAFIMO: Variant and haplotype aware motif scanning on pangenome graphs
Source: PLoS Comput Biol. 2021 Sep 27;17(9):e1009444. doi: 10.1371/journal.pcbi.1009444 (PMC8519448; doi:10.1371/journal.pcbi.1009444)
Supplement: S1 Text — Fig A. Example of TSV summary report. The tab-delimited report (TSV report) shows the first 25 potential CTCF occurrences retrieved by GRAFIMO, searching the motif in ChIP-seq peak regions defined in ENCODE experiment ENCFF816XLT (cell line A549). Fig B. Example of HTML summary report. The HTML report shows the first 25 potential CTCF occurrences retrieved by GRAFIMO, searching the motif in ChIP-seq peak regions defined in ENCODE experiment ENCFF816XLY (cell line A549). Fig C. Example of GFF3 track produced by GRAFIMO, loaded on the UCSC genome browser. GRAFIMO returns also a GFF3 report which can be loaded on the UCSC genome browser; the loaded custom track shows three potential CTCF occurrences (region chr8:142,782,661–142,782,680) retrieved by GRAFIMO overlapping a dbSNP annotated variant (rs892844) (image obtained from the UCSC Genome Browser website). Fig D. Structure of transcription factor motifs used to test GRAFIMO. Transcription factor binding site motifs of (A) CTCF, (B) ATF3 and (C) GATA1. Fig E. Searching ATF3 motif on VG with GRAFIMO provides an insight on how genetic variation affects the binding site sequence. (A) Potential ATF3 occurrences statistically significant (P-value < 1e-4) and non-significant found in the reference and in the haplotype sequences found with GRAFIMO oh hg38 1000GP VG. (B) Statistical significance of retrieved potential ATF3 motif occurrences and their frequency in the haplotypes embedded in the VG. (C) Percentage of statistically significant ATF3 potential binding sites found only in genome reference sequence, percentage of potential TFBS found in the reference for which genetic variants cause the sequence to be no more significant, percentage of binding sites found only in the haplotypes, percentage of potential TFBS found in the reference with increased statistical significance by the action of genomic variants and percentage of those with a decreased significance by the action of variants (with P-value still significant). [file pcbi.1009444.s001.docx]

**Supplementary materials of “GRAFIMO: variant and haplotype aware motif scanning on pangenome graphs”**

**Manuel Tognon^1^, Vincenzo Bonnici^1^, Erik Garrison^2^, Rosalba Giugno^1 *^, Luca Pinello^3,4,5 *^**

**1** Computer Science Department, University of Verona, Verona, Italy

**2** University of Tennessee Health Science Center, Memphis, Tennessee, United States of America

**3** Molecular Pathology Unit, Center for Computational and Integrative Biology and Center for Cancer Research, Massachusetts General Hospital, Charlestown, Massachusetts, United States of America

**4** Department of Pathology, Harvard Medical School, Boston, Massachusetts, United States of America

**5** Broad Institute of MIT and Harvard, Cambridge, Massachusetts, United States of America

*lpinello@mgh.harvard.edu; *rosalba.giugno@univr.it

**1. 1000 Genomes genomic variants**

To validate GRAFIMO, we tested it on a pangenome variation graph (VG) [1] based on 2548 individuals from the 1000 Genomes Project (1000GP) phase 3 on GRCh38 cohort [2,3], encoding all their genomic variants and their haplotypes. We constructed the 1000GP VG using ~78 millions of genomic variants (SNPs and indels) (see **Table A** in **S1 Text**), belonging to the 2548 considered subjects and constituted a total of 5096 haplotypes.

**2. GRAFIMO on ENCODE Project’s ChIP-seq data**

To test GRAFIMO we selected three transcription factor (TF) motifs of different length, evolutionary conservation, information content, and tolerance to point mutations: CTCF (**Fig D (A)** in **S1 Text**), ATF3 (**Fig D (B)** in **S1 Text**) and GATA1 (**Fig D (C)** in **S1 Text**). For each TF we obtained ChIP-seq optimal IDR thresholded peak regions (in ENCODE bigBED format) from the ENCODE Project database [4,5]. The ChIP-seq peaks were mapped on the hg38 human genome assembly and were retrieved from different cell types (see **Table B** in **S1 Text**). To obtain a suitable input for GRAFIMO, the bigBED files were converted to the corresponding BED files with UCSC bigBedToBed tool [6]. The resulting BED files were filtered in order to contain only features related to the canonical chromosomes and were sorted by *q*-values to select the most informative regions (top 3000 for each experiment).

**3. Searching for CTCF occurrences**

CTCF is a zinc-finger transcription factor involved in transcriptional regulation, which plays a fundamental role in epigenetic regulation [7] and acts as tumor suppressor [8]. During our tests, we searched CTCF motif (**Fig D (A)** in **S1 Text**) (JASPAR ID MA0139.1) on the 1000GP VG (hg38 genome assembly) (see **S1 Text section 1**). To have binding events likely to happen, CTCF motif has been searched on regions corresponding to the top 3000 significant ChIP-seq peaks regions (sorted and filtered by *q*-value, see **S1 Text section 2**) for CTCF on the A549, HepG2, GM12878, K562, and MCF-7 cell lines (see **Table B** in **S1 Text**).

**4. Searching for ATF3 occurrences**

Activating Transcription Factor 3 (ATF3) is a transcription factor belonging to the family of cAMP responsive element-binding. Its activity is induced by physiological stress in a wide variety of tissues [9] and has been shown to have significant roles in both immunity and cancer [10]. ATF3 binds short, conserved DNA sequences. We searched the ATF3 motif (**Fig D (B)** in **S1 Text**) (JASPAR ID MA0605.2) on a hg38 VG enriched with SNPs and indels from 2548 individuals of 1000GP phase 3. As done for CTCF, to have likely to happen binding events we searched ATF3 motif on regions corresponding to the top 3000 significant ChIP-seq optimal IDR thresholded peaks (sorted and filtered by *q*-value, see **S1 Text section 2**) for ATF3 on the H1, HepG2 and K562 cell lines (see **Table B** in **S1 Text**).

For our downstream analysis we selected only the ATF3 motif occurrence whose *P-*value was < 1e^-4^ and we considered them as potential binding sites. We found several potential motif occurrences which would be lost scanning only the reference genome sequence (**Fig E (A)** in **S1 text**). Moreover, we observed that many ATF3 motif candidates with highly statistically significant *P*-values are found in rare haplotypes (**Fig E (B)** in **S1 Text**). We also found that 7.03% of the potential ATF3 binding sites can be found only in non-reference haplotype sequences, 11.28% are disrupted by genomic variants in non-reference haplotypes and ~13% of the potential ATF3 TFBS are still significant in non-reference haplotypes but showing different binding scores (**Fig E (C)** in **S1 Text**). We also observed that a considerable fraction of putative ATF3 binding sites are population specific. In fact, 19.81%, 3.77%, 7.55%, 19.81%, 19.81% of potential TFBS retrieved on individual haplotypes are specific for AFR, EUR, AMR, SAS and EAS populations, respectively (**Fig E (D)** in **S1 Text**).

**5. Searching for GATA1 occurrences**

GATA1 is a zinc-finger transcription factor having a fundamental role during the development of hematopoietic cell lineages [11]. GATA1 binds short (11 bp) highly conserved DNA sequences. GATA1 motif (**Fig D (C)** in **S1 Text**) (JASPAR ID MA0035.4) has been searched with GRAFIMO on a hg38 VG enriched with SNPs and indels from 2548 individuals of 1000GP phase 3. In order to have likely to happen binding events, we searched GATA1 motif occurrences in regions corresponding to the top 3000 significant ChIP-seq optimal IDR thresholded peaks (sorted and filtered by *q*-value, see **S1 Text section 2**) for GATA1 on the K562 cell line (see **Table B in S1 Text**).

In our downstream analysis we considered only the motif occurrences, whose *P*-value was < 1e^-4^ and we considered them as potential binding sites. We observed that many potential GATA1 occurrences are lost when searching the motif only in the reference genome (**Fig F (A)** in **S1 Text**). We also found that several potential motif occurrences with a highly statistically significant *P*-value are detected in rare haplotypes (**Fig F (B)** in **S1 Text**). Moreover, we found that 9.78% of the potential GATA1 TFBS can be found only in non-reference haplotype sequences, 12.58% are disrupted by genomic variants and ~4% are still significant in non-reference haplotypes but with different binding scores (**Fig F (C)** in **S1 Text**). We also found that several potential GATA1 binding sites among those retrieved only in individual genome sequences are population specific. We observed that 25.97% of putative binding sites are specific for AFR, 3.90% for EUR, 9.09% for AMR, 19.48% for SAS and 11.69% for EAS populations (**Fig F (D)** in **S1 Text**).

**6. Comparing FIMO and GRAFIMO**

To assess GRAFIMO correctness we compared the obtained results with those retrieved running FIMO [12] on the same ChIP-seq regions used to test our tool. We run both GRAFIMO and FIMO on a Linux-based machine (OS Ubuntu 18.04), with an Intel(R) Core (TM) i7- 5960X 3.00GHz CPU (16 cores) and 64GB of RAM. FIMO requires in input a set of sequences given via a FASTA file. For each tested TF motif, we obtained the reference genome sequences corresponding to the ChIP-seq optimal IDR thresholded peaks used to test GRAFIMO using BEDTools [13] *getfasta* functionality. We observed that, for each TF motif, GRAFIMO does not lose any potential motif occurrence with respect to those retrieved running FIMO. Thus, GRAFIMO detects more motif occurrence candidates, located on the alternative haplotypes embedded in the VG, without losing any potential binding sites in the reference genome sequence.

We also benchmarked GRAFIMO comparing its running time and memory usage to FIMO. To this end we searched for potential occurrences of the CTCF motif (19 bp wide, JASPAR ID MA0139.1) on forward and reverse strand in 1000 genomic regions of the human chr22 of increasing length (1 to 9 million). To run FIMO, for each set of genomic regions we created FASTA file as required by this tool.

We assessed GRAFIMO performance scanning the chr22 VG without any encoded genetic variant for CTCF motif occurrences, on the previously computed sets of genomic regions. Moreover, since FIMO does not provide a parallel implementation, we run GRAFIMO using a single thread. In this scenario FIMO is faster and requires less memory than GRAFIMO (**Fig H** in **S1 Text**).

We also benchmarked GRAFIMO running time with a single thread when performing a motif search on the VG encoded chr22 with 1000GP genetic variants (2548 individuals). In this test setting, excluding the preprocessing required to retrieve the FASTA genomic sequences for each of the 2548 1000GP subjects from the running time calculations, GRAFIMO proves to be faster, although FIMO speed is similar scanning regions up to 1 million of bp (**Fig H** **(C)** in **S1 Text** ).

These results are expected since FIMO is a highly optimized tool to scan linear genomic sequences, while GRAFIMO has been designed to efficiently scan panels of individuals simultaneously and to run in parallel using multiple threads.

We also assessed GRAFIMO running time and memory usage when using 1, 4, 8 and 16 threads, respectively. This analysis was performed using the chr22 VG with 1000GP genetic variants (**Fig I** in **S1 Text**). As expected, running the tool with multiple threads resulted in a dramatical reduction of the running time, however the memory usage remains similar increasing the number of threads used. Not surprisingly, running GRAFIMO in parallel on the chr22 VG enriched with variants produces the major improvements in running time.

To obtain the results presented in the manuscript on average each scan required ~15 minutes and used ~24 GB of memory.

**7. Installing and running GRAFIMO**

In this section will be presented how to install and run GRAFIMO. For further details on installation and run refer to GRAFIMO’s README and Wiki at <https://github.com/pinellolab/GRAFIMO> and <https://github.com/InfOmics/GRAFIMO>.

Before installing GRAFIMO the user must have installed:

- VG, v1.27.1 or later (https://github.com/vgteam/vg)
- Tabix (https://github.com/samtools/htslib)
- Graphviz (https://graphviz.org)

GRAFIMO has been written in Python3 and Cython. To build GRAFIMO, Cython is required to be installed. To build GRAFIMO are also used *Setuptools* and *Wheel* Python packages. Moreover, GRAFIMO depends on the following Python packages:

- *Pandas*
- *NumPy*
- *Statsmodels*
- *Sphinx*
- *Numba*
- *Colorama*

Once all the dependencies have been satisfied, GRAFIMO can be installed via pip typing on terminal:

pip3 install grafimo

GRAFIMO can also be built from source code, by cloning GRAFIMO repository on Github. To clone the repository, type:

git clone https://github.com/pinellolab/GRAFIMO.git.

To build GRAFIMO, type:

cd GRAFIMO; python3 setup.py install –user

To install GRAFIMO via Bioconda (for Linux users only), type:

conda install grafimo

To scan a pangenome variation graph with GRAFIMO are required the path to a directory containing the VGs of all the chromosome (XG and GBWT indexes) or the path to a whole pangenome variation graph (note that the XG and GBWT index of the VG must be stored in the same location), a motif PWM given in JASPAR or MEME format and a BED file containing the genomic regions where the motif will be searched.

Let us assume that we built the pangenome variation graph by constructing a VG for each chromosome. To find potential motif occurrences in the VG, type

grafimo -d /path/to/directory/storing/my/graphs/ -b /path/to/my/bedfile -m path/to/my/motif

To scan a whole pangenome variation graph for the occurrence of the given motif, type

grafimo -g /path/to/my/whole/genome/vg -b /path/to/my/bedfile -m /path/to/my/motif

With GRAFIMO it is also possible to build a pangenome variation graph from user data. To construct a VG are required a FASTA file containing the reference genome and a VCF file containing the phased genomic variants to enrich the reference sequence.

To build the pangenome variation graph with GRAFIMO, type

grafimo buildvg -l /path/to/reference/genome -v /path/to/vcf/file

Hands-on tutorials on how to run GRAFIMO are available at <https://github.com/pinellolab/GRAFIMO> and <https://github.com/InfOmics/GRAFIMO>.

**
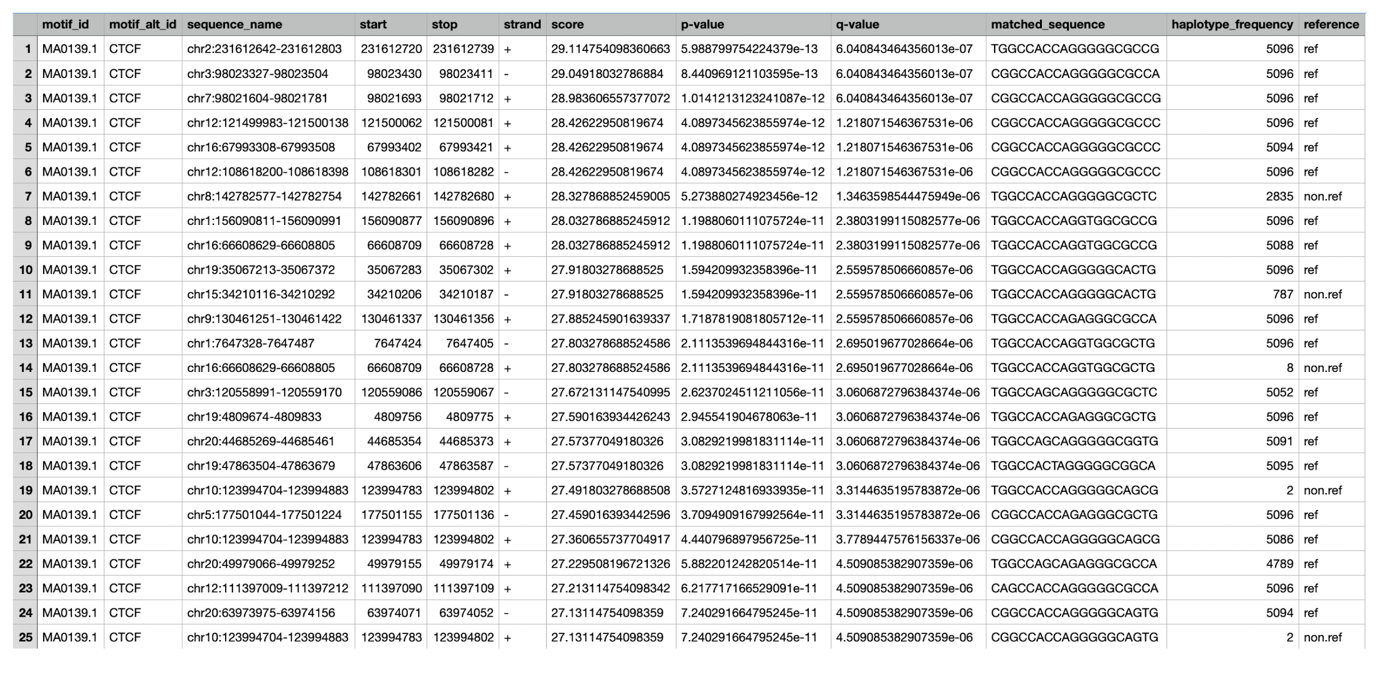
**

**Fig A. Example of TSV summary report.** The tab-delimited report (TSV report) shows the first 25 potential CTCF occurrences retrieved by GRAFIMO, searching the motif in ChIP-seq peak regions defined in ENCODE experiment ENCFF816XLT (cell line A549).

**
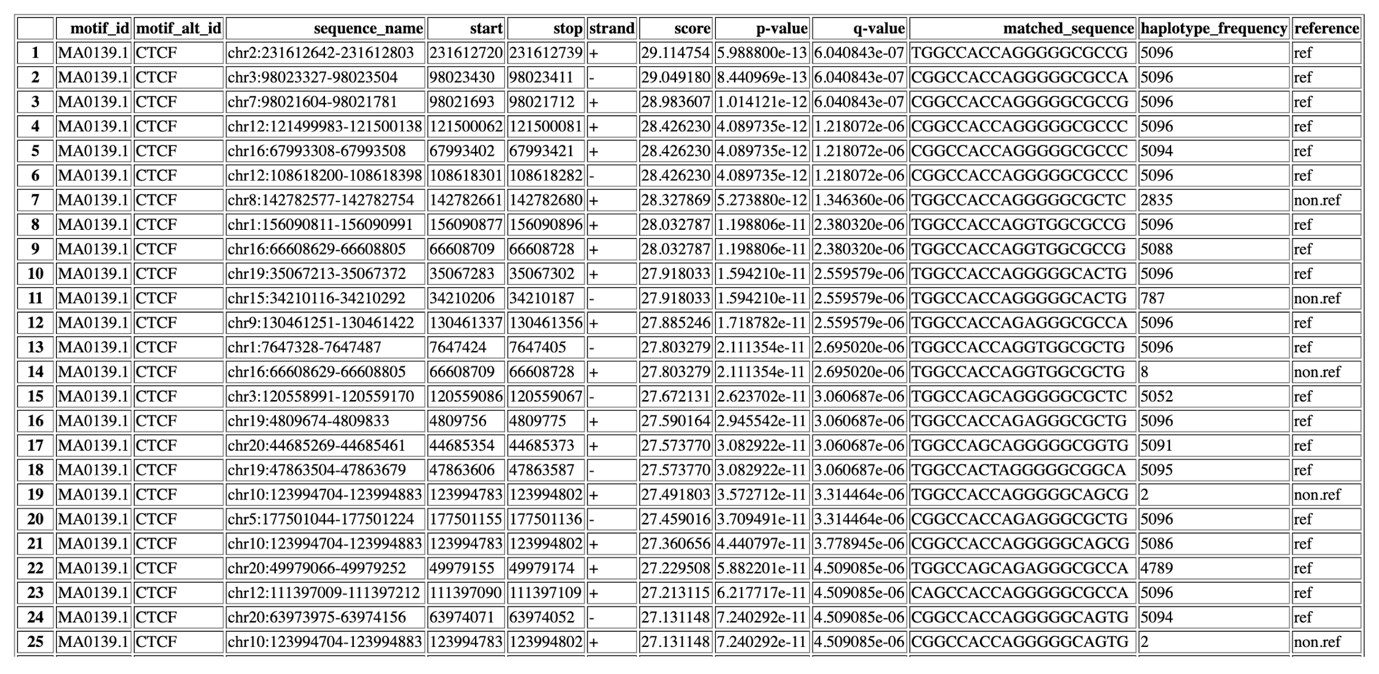
**

**Fig B. Example of HTML summary report.** The HTML report shows the first 25 potential CTCF occurrences retrieved by GRAFIMO, searching the motif in ChIP-seq peak regions defined in ENCODE experiment ENCFF816XLY (cell line A549).

**
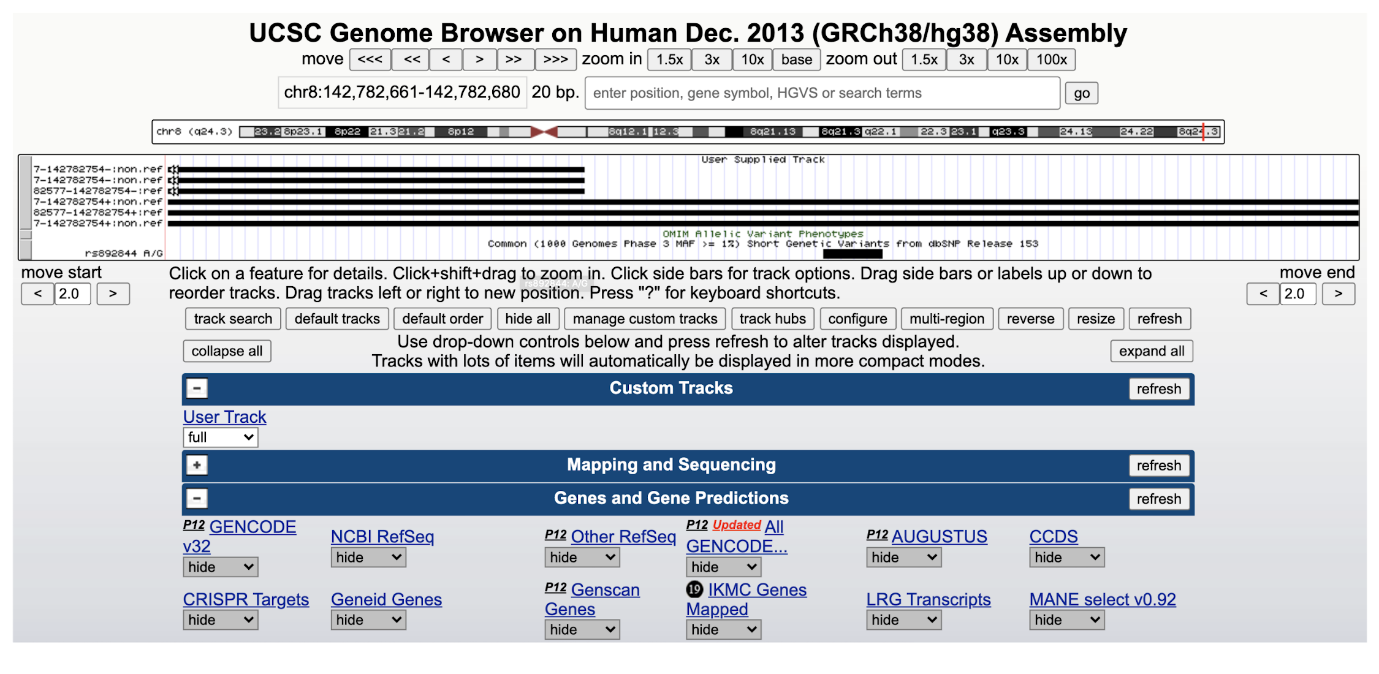
**

**Fig C. Example of GFF3 track produced by GRAFIMO, loaded on the UCSC genome browser.** GRAFIMO returns also a GFF3 report which can be loaded on the UCSC genome browser; the loaded custom track shows three potential CTCF occurrences (region chr8:142,782,661-142,782,680) retrieved by GRAFIMO overlapping a dbSNP annotated variant (rs892844) (image obtained from the UCSC Genome Browser website [14]).

**
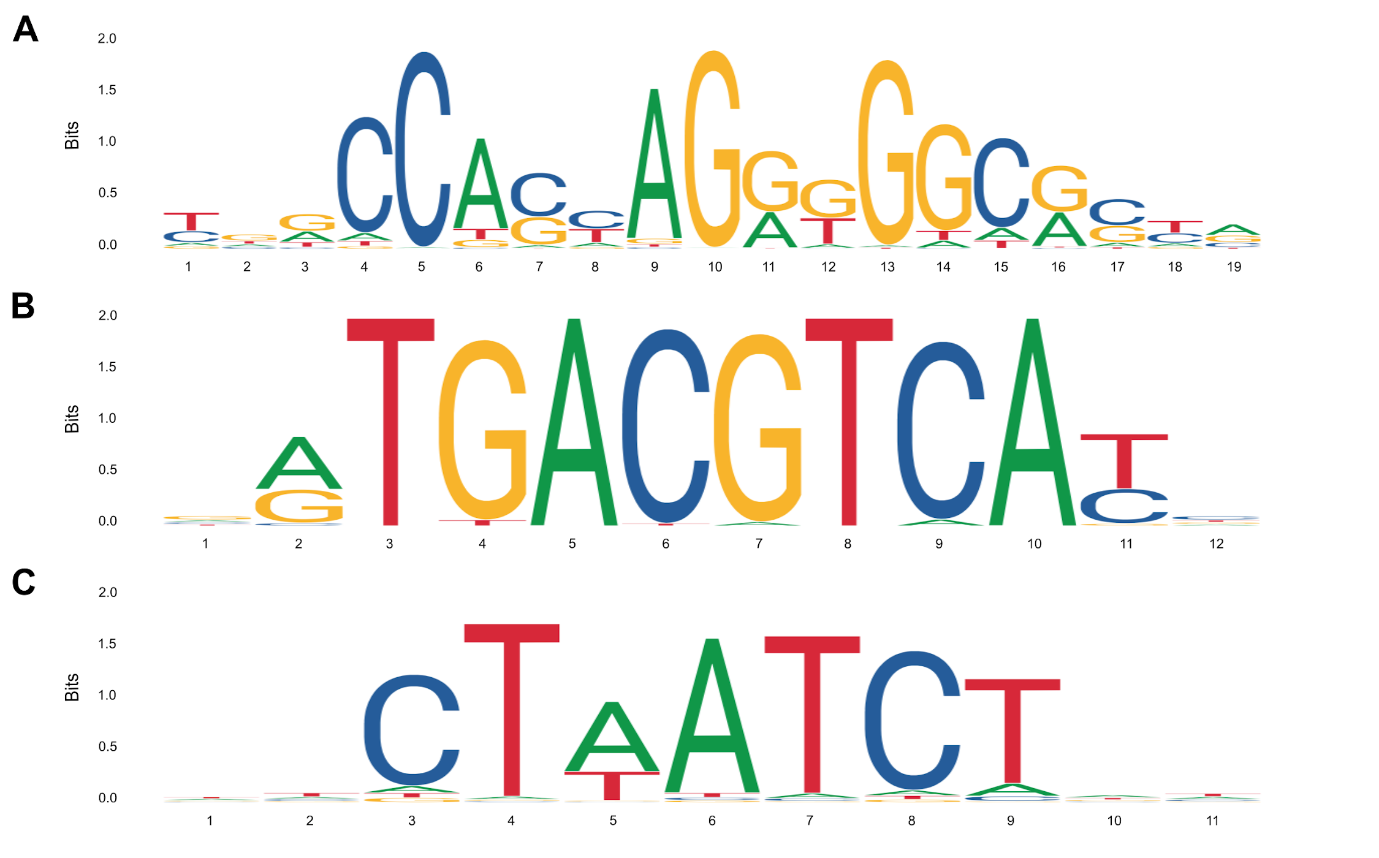
**

**Fig D. Structure of transcription factor motifs used to test GRAFIMO.** Transcription factor binding site motifs of (A) CTCF, (B) ATF3 and (C) GATA1.

**
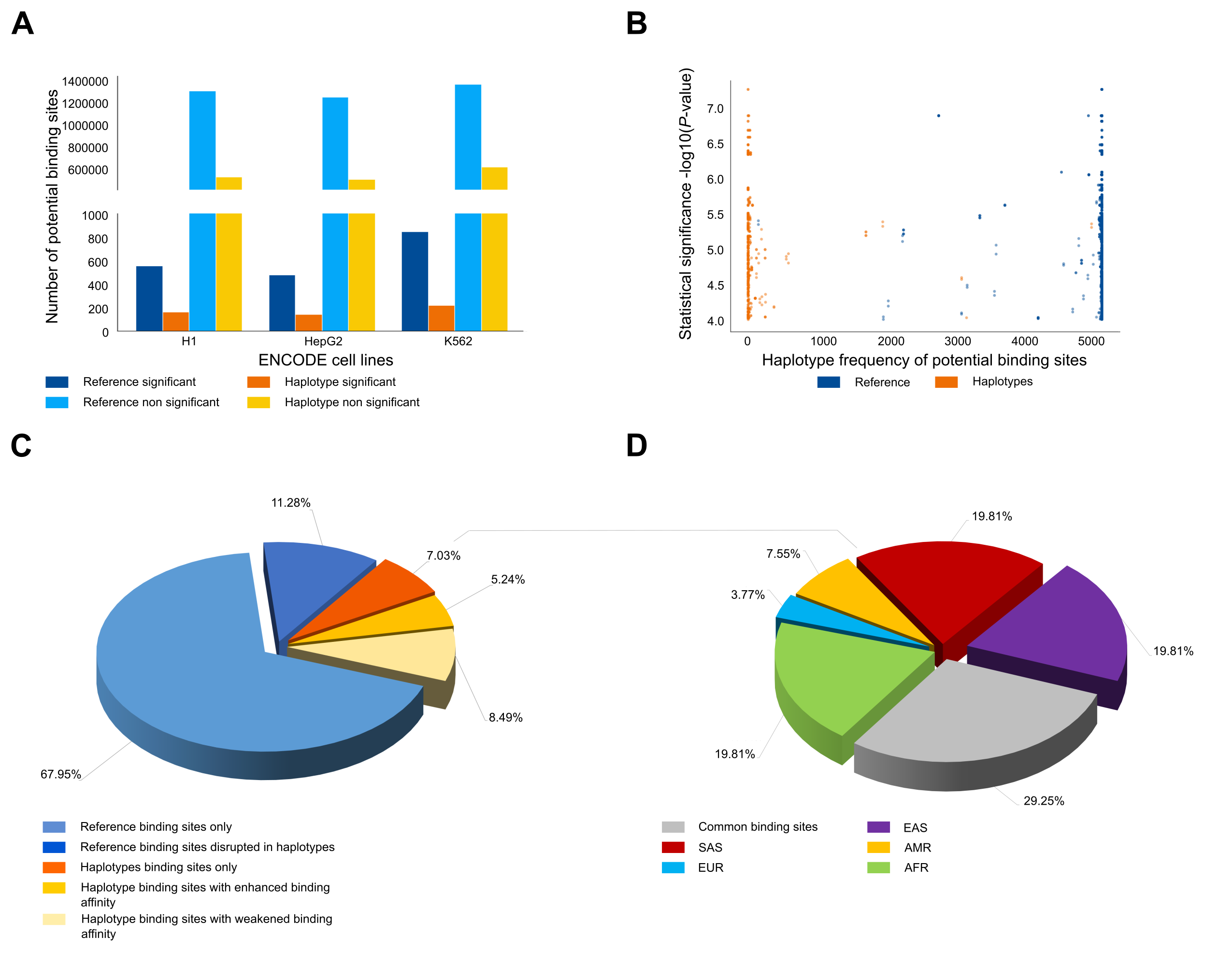
**

**Fig E. Searching ATF3 motif on VG with GRAFIMO provides an insight on how genetic variation affects putative binding sites**. (A) Potential ATF3 occurrences statistically significant (P-value < 1e-4) and non-significant found in the reference and in the haplotype sequences found with GRAFIMO oh hg38 1000GP VG. (B) Statistical significance of retrieved potential ATF3 motif occurrences and their frequency in the haplotypes embedded in the VG. (C) Percentage of statistically significant ATF3 potential binding sites found only in the reference genome or alternative haplotypes and with modulated binding scores based on 1000GP genetic variants. (D) Percentage of population specific and common (shared by two or more populations) potential ATF3 binding sites present on individual haplotypes.

**
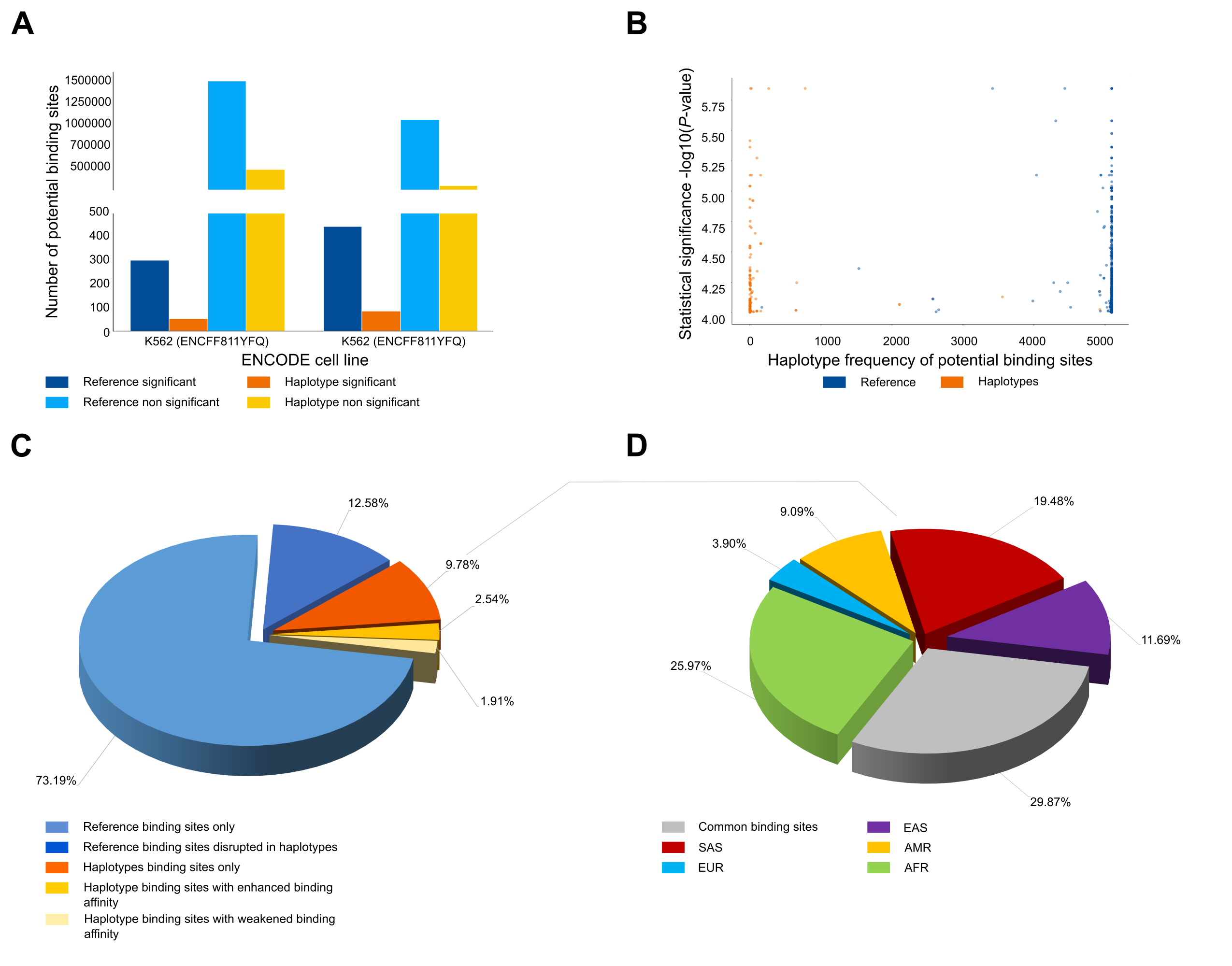
**

**Fig F. Searching GATA1 motif on VG with GRAFIMO provides an insight on how genetic variation affects putative binding sites.** (A) Potential GATA1 occurrences statistically significant (P-value < 1e-4) and non-significant found in the reference and in the haplotype sequences found with GRAFIMO oh hg38 1000GP VG. (B) Statistical significance of retrieved potential GATA1 motif occurrences and their frequency in the haplotypes embedded in the VG. (C). Percentage of statistically significant GATA1 potential binding sites found only in the reference genome or alternative haplotypes and with modulated binding scores based on 1000GP genetic variants. (D) Percentage of population specific and common (shared by two or more populations) potential GATA1 binding sites present on individual haplotypes.

**
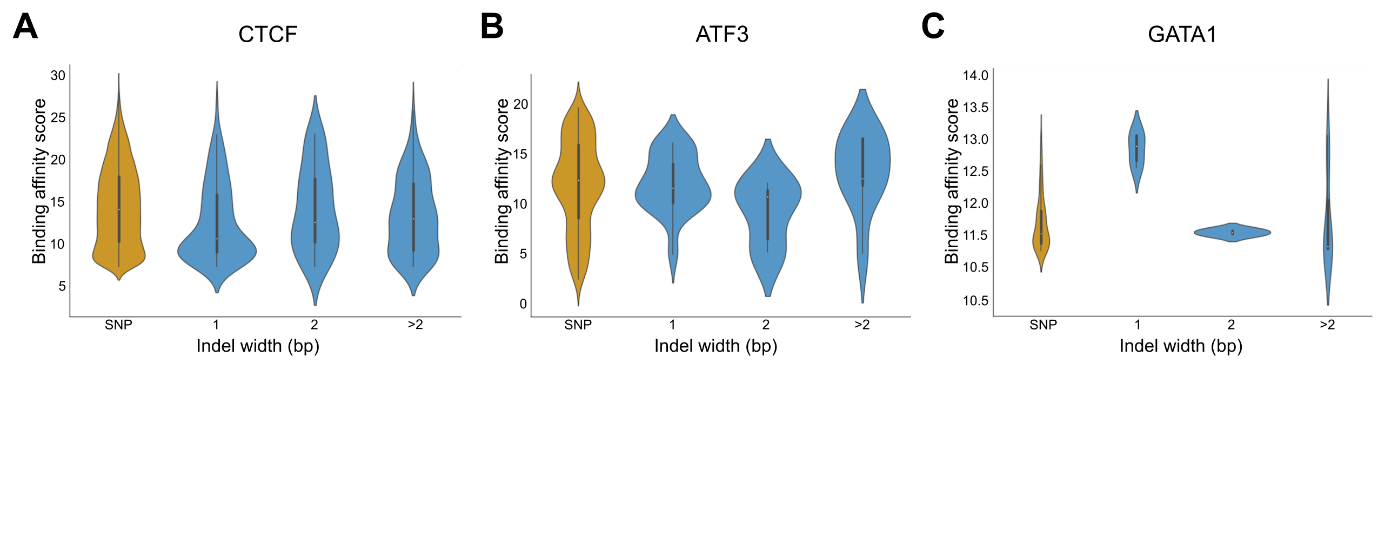
**

**Fig G. Influence of different type of mutations and length on binding affinity score**. (A) CTCF, (B) ATF3, (C) GATA1.

**
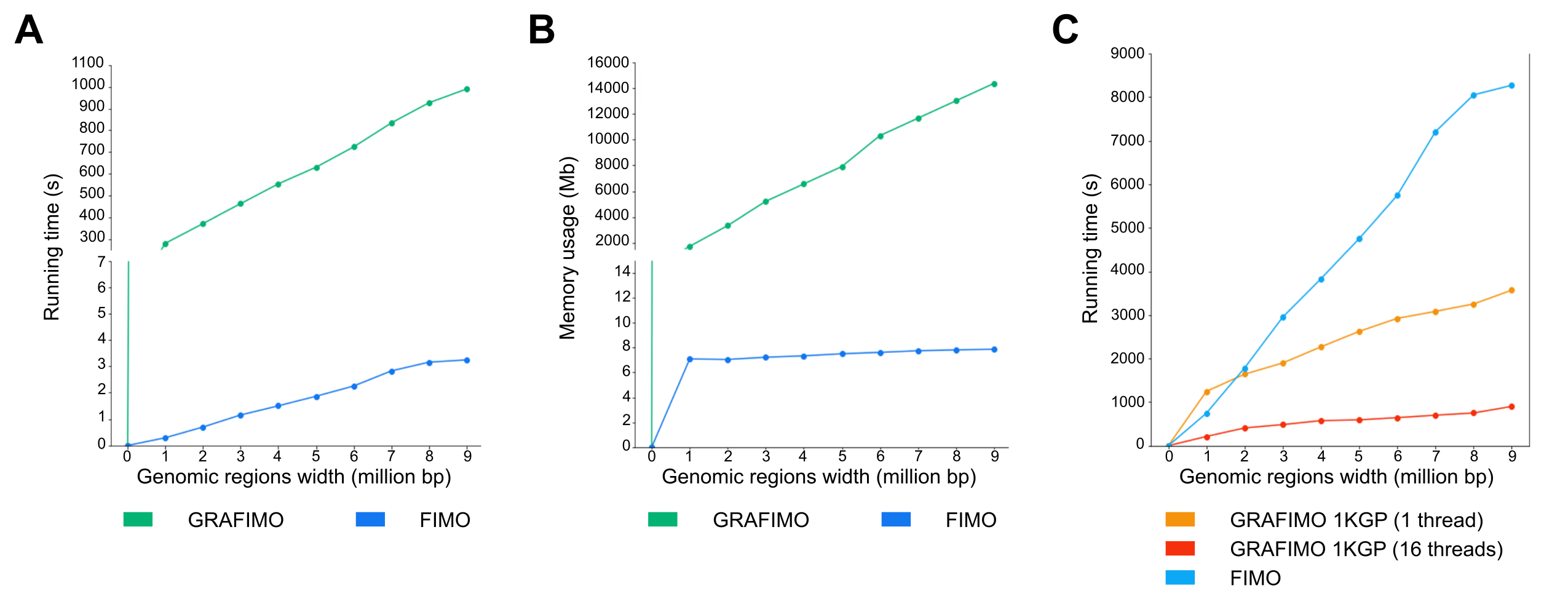
**

**Fig H. Comparing GRAFIMO and FIMO performance.** (A) FIMO is faster than GRAFIMO (using a single thread) when searching CTCF motif (JASPAR ID MA0139.1) on human chr22 regions (total width ranging from 1 to 9 millions of bp) and without accounting for genetic variants. (B) FIMO uses less memory resources than GRAFIMO, however it can only scan linear sequences. (C) GRAFIMO is generally faster than FIMO in searching potential CTCF occurrences after 1Mbp when considering genetic variation present in large panels of individuals as 1000GP on GRCh38 phase 3 (2548 samples), even with a single thread.


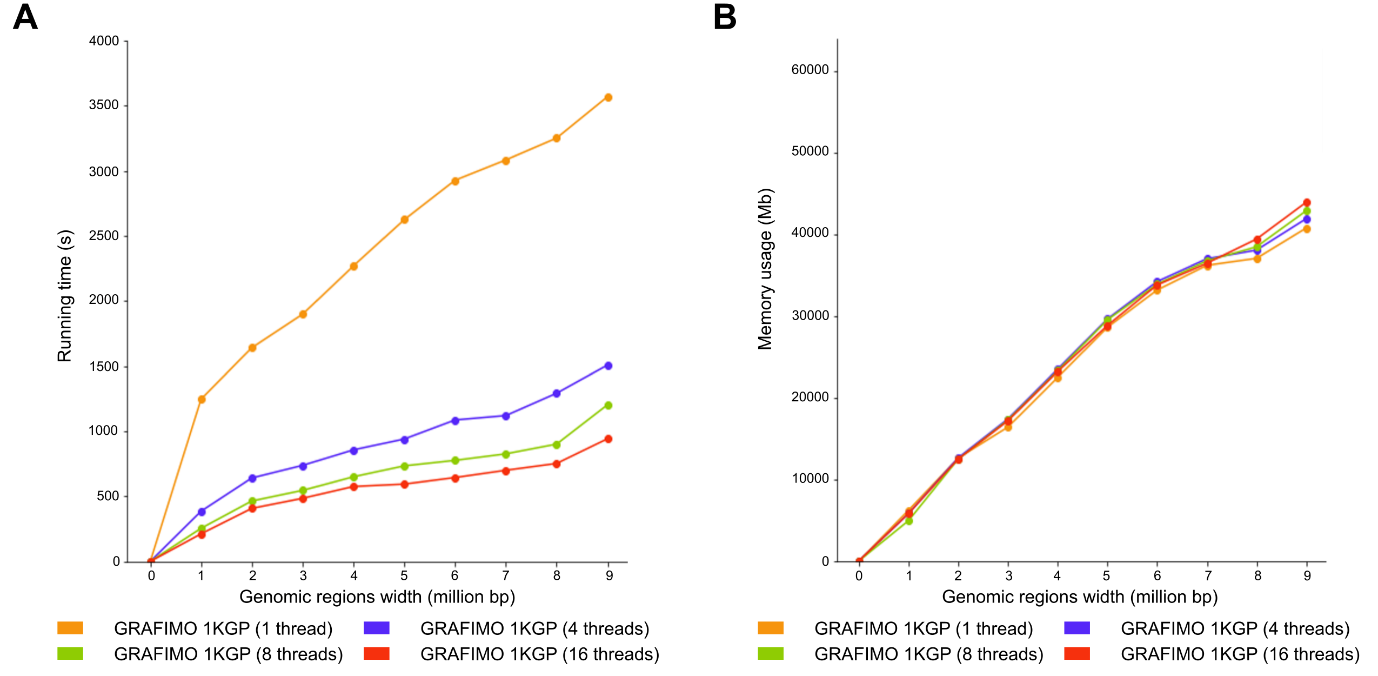


**Fig I. GRAFIMO running time efficiently scales with** **the number of threads used.** By running GRAFIMO with multiple threads (A) the running time significantly decreases, while (B) memory usage remains similar.

**Table A.** **Number of genomic variants used to test GRAFIMO.** Number of genomic variants used to test GRAFIMO, divided by chromosome. The variants were obtained from 1000 Genomes Project on GRCh38 phase 3, and belongs to 2548 individuals from 26 populations. The number of variants refers to SNPs and indels together. In total were considered ~78 million variants.

| **Chromosome** | **Number of SNPs and indels** |
| --- | --- |
| **Chr1** | 6,191,833 |
| **Chr2** | 6,790,551 |
| **Chr3** | 5,641,493 |
| **Chr4** | 5,477,810 |
| **Chr5** | 5,115,036 |
| **Chr6** | 4,863,337 |
| **Chr7** | 4,511,408 |
| **Chr8** | 4,425,449 |
| **Chr9** | 3,384,360 |
| **Chr10** | 3,874,259 |
| **Chr11** | 3,881,791 |
| **Chr12** | 3,745,465 |
| **Chr13** | 2,760,845 |
| **Chr14** | 2,548,903 |
| **Chr15** | 2,301,453 |
| **Chr16** | 2,548,920 |
| **Chr17** | 2,209,149 |
| **Chr18** | 2,189,529 |
| **Chr19** | 1,738,824 |
| **Chr20** | 1,817,492 |
| **Chr21** | 1,045,269 |
| **Chr22** | 1,059,079 |
| **ChrX** | 106,963 |

**Table B. ENCODE ChIP-seq experiment codes.** To test our software, we searched the potential occurrences of three transcription factor motifs (CTCF, ATF3 and GATA1) in a hg38 pangenome variation graph enriched with genomic variants and haplotypes of 2548 individuals from 1000 Genomes project phase 3. To have likely to happen binding events, TF motifs were searched in ChIP-seq peak regions, obtained from the ENCODE project data portal.

| **Motif** | **Cell line A549** | **Cell line GM12878** | **Cell line H1** | **Cell line HepG2** | **Cell line K562** | **Cell line MCF-7** |
| --- | --- | --- | --- | --- | --- | --- |
| **CTCF** | ENCFF816XLT | ENCFF267NYF |  | ENCFF015OJG | ENCFF895HAG | ENCFF088JWU |
| **ATF3** |  |  | ENCFF207AVV | ENCFF753WNT | ENCFF787GVU |  |
| **GATA1** |  |  |  |  | ENCFF811YFQ |  |
|  |  |  |  |  | ENCFF939ODZ |  |

**References**

1. Garrison E, Sirén J, Novak AM, Hickey G, Eizenga JM, Dawson ET, et al. Variation graph toolkit improves read mapping by representing genetic variation in the reference. Nature biotechnology. 2018;36(9): 875—879.
2. Zheng-Bradley X, Streeter I, Fairley S, Richardson D, Clarke L, Flicek P, et al. Alignment of 1000 Genomes Project reads to reference assembly GRCh38. GigaScience. 2017;6(7): 1—8.
3. Lowy-Gallego E, Fairley S, Zheng-Bradley X, Ruffier M, Clarke L, Flicek P*.* Variant Calling on the GRCh38 assembly with the data from phase three of the 1000 Genomes Project. Wellcome Open Research, 2019;4.
4. ENCODE Project Consortium. An Integrated encyclopedia of DNA elements in the human genome. Nature. 2012;489(7414): 57—74.
5. Davis CA, Hitz BC, Sloan CA, Chan ET, Davidson JM, Gabdank I, et al. The encyclopedia of DNA elements (ENCODE): data portal update. Nucleic Acid Research. 2018;46(D1): D794—D801.
6. Kent WJ, Zweig AS, Barber G, Hinrichs AS, Karolchik D*.* BigWig and BigBed: enabling browsing of large distributed datasets. Bioinformatics. 2010;26(17): 2204—2207.
7. Ishihara K, Oshimura M, Nakao M. CTCF-dependent chromatin insulator is linked to epigenetic remodeling. Molecular Cell. 2006;23(5): 733—742.
8. Fiorentino FP, Giordano A. The tumor suppressor role of CTCF. Journal of cellular physiology. 2012;227(2): 479—492.
9. Chen BP, Wolfgang CD, Hai T. Analysis of ATF3, a transcription factor induced by physiological stresses and modulated by gadd153/Chop10. Molecular and cellular biology. 1996;16(3): 1157—1168.
10. Thompson MR, Xu D, Williams BRG. ATF3 Transcription factor and its emerging roles in immunity and cancer. Journal of molecular medicine. 2009;87(11): 1053.
11. Calligaris R, Bottardi S, Cogoi S, Apezteguia I, Santoro C*.* Alternative translation initiation site usage results in two functionally distinct forms of the gata-1 transcription factor. Proceedings of the National Academy of Sciences. 1995;92(25): 11598—11602.
12. Grant CE, Bailey TL, Noble WS*.* Fimo: scanning for occurrences of a given motif. Bioinformatics. 2011;27(7): 1017—1018.
13. Quinlan AR, Hall IM. BEDTools: a flexible suite of utilities for comparing genomic features. Bioinformatics. 2010;26(6): 841—842.
14. Lee CM, Barber GP, Casper J, Clawson H, Diekhans M, Navarro Gonzalez J, et al. UCSC Genome Browser enters 20th year. Nucleic Acid Research. 2020;48(D1): D756–D761.
