# Supplementary figures and images for: GRAFIMO: Variant and haplotype aware motif scanning on pangenome graphs
Source: PLoS Comput Biol. 2021 Sep 27;17(9):e1009444. doi: 10.1371/journal.pcbi.1009444 (PMC8519448; doi:10.1371/journal.pcbi.1009444)

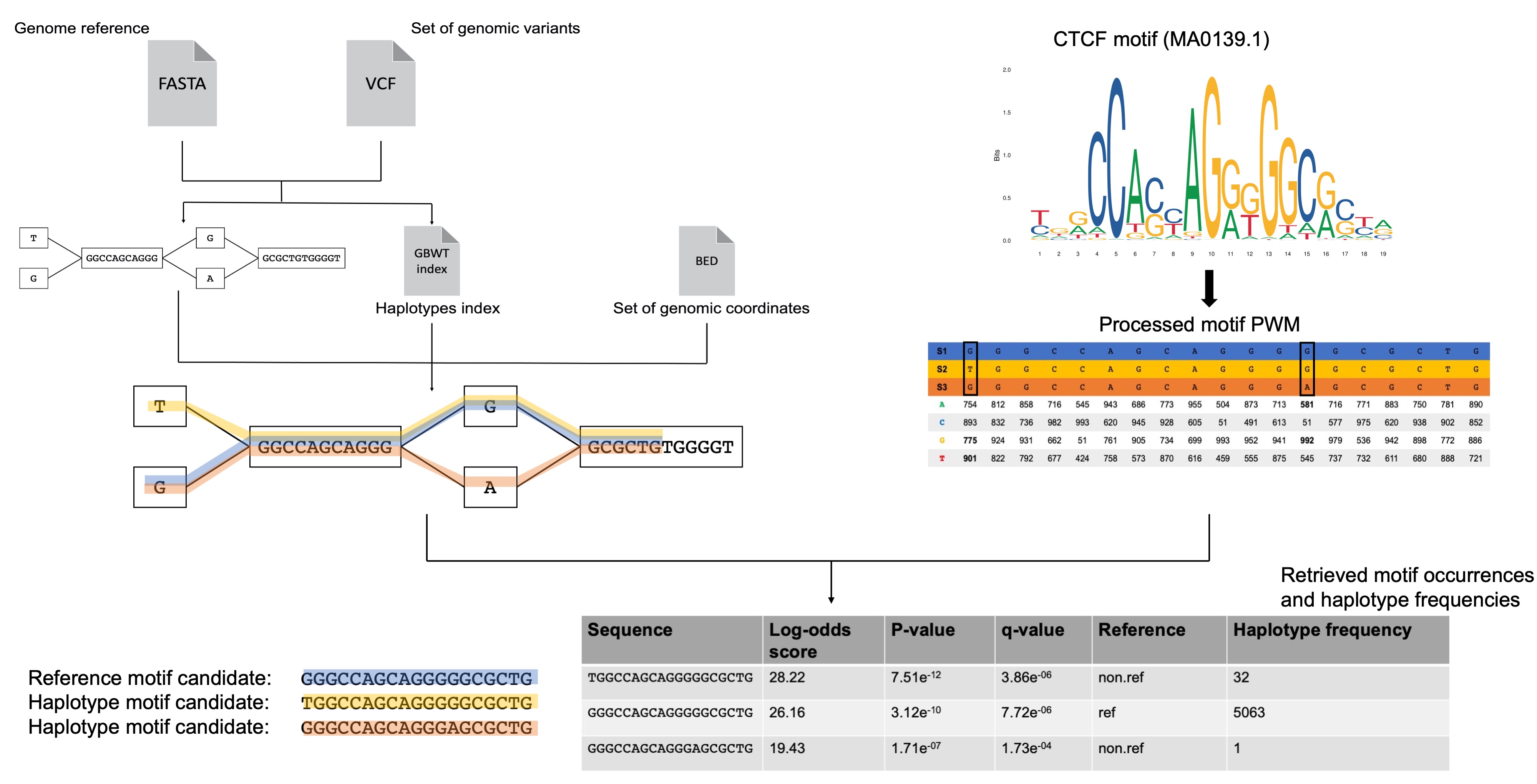

Supplement: S1 Code — (ZIP) [file pcbi.1009444.s002.zip › S1_code/GRAFIMO/docs/wf.jpg]

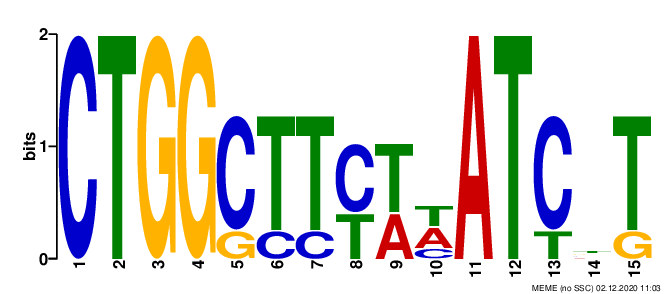

Supplement: S1 Code — (ZIP) [file pcbi.1009444.s002.zip › S1_code/GRAFIMO/tutorials/findmotif_tutorial/data/example.png]
