# Supplementary material for: GRAFIMO: Variant and haplotype aware motif scanning on pangenome graphs
Source: PLoS Comput Biol. 2021 Sep 27;17(9):e1009444. doi: 10.1371/journal.pcbi.1009444 (PMC8519448; doi:10.1371/journal.pcbi.1009444)
Supplement: Attachment — Submitted filename: Response_to_reviewers.pdf [file pcbi.1009444.s004.pdf]

Response to reviewers

Title: “GRAFIMO: variant and haplotype aware motif scanning via pangenome graphs”
ID: PCOMPBIOL-D-21-00041
Authors: Manuel Tognon, Vincenzo Bonnici, Erik Garrison, Rosalba Giugno and Luca Pinello

We would like to thank the reviewers for the positive comments and feedback. We believe that we have addressed all the recommendations pointed out by the reviewers and the suggestions helped to furtherly improve the manuscript.
We provide below a point-by-point response. The changes to the manuscript were highlighted in red.

Reviewer 1

1) I finished reviewing the revised manuscript. The authors have answered all my previously proposed questions, and the work is ready to be accepted. It’s a nice tool and will potentially have many users!

We are glad the revised manuscript addressed all the points raised before. We also want to thank the reviewer for the several suggestions that significantly improved the manuscript and the software.

Reviewer 2

1) I think the experiments you have performed for examining running time and memory usage are satisfactory. In the end of the section titled "Transcription factor motif search" you have added a sentence referring to these experiments. I find it a bit weird to refer to the experiments here. Wouldn't this be better suited for the Results section? The way you phrase this sentence is also a bit vague. You say "S1 Text section 6 describes how the tool performance compares to those of a tool designed to work on a single genomic sequence at a time, such as FIMO". I think two things could be improved here: 1) You could just say that you compare the performance to Fimo, instead of "tools, such as FIMO" (since you only compare to FIMO) and 2) it would be nice if you could very briefly summarize the main findings here, instead of just referring to the supplementary. What I would prefer is a phrasing along the lines of "We also compared the performance of GRAFIMO against FIMO, and found that ... (see S1 Text Section 6).".

We kindly thank the reviewer for the suggestions and modified the manuscript accordingly. We moved running time and memory usage experiments description to the end of **Results** section, and we briefly described the obtained results.

The manuscript was extended by adding this new text at the end of **Results** section:

*“We also compared the performance of GRAFIMO against FIMO [13] (**S1 Text Section 6** and **S8 Fig**). FIMO is faster and requires less memory, when scanning a single linear genome. However, when considering the 2548 individual genomes and their genetic variation, GRAFIMO proves to be generally faster than FIMO. Moreover, we benchmarked how GRAFIMO running time and memory usage change using an increasing number of threads (**S9 Fig**). By increasing the number of threads, we observed a dramatical drop in running time, while memory usage remained similar.”*

2) In the caption of S9 Figure you say that memory usage increases when using multiple threads. But from the figure, it seems that the memory usage is about the same for all the cases. Am I right or have I misunderstood the figure?

We thank the reviewer for raising this point. We agree with the reviewer that by using different number of threads the memory usage is similar.

Therefore, we highlighted this observation in **S1 Text section 6** as follows:

"As expected, running the tool with multiple threads resulted in a dramatical reduction of the running time, ***however the memory usage remains similar increasing the number of threads used****. Not surprisingly, running GRAFIMO in parallel on the chr22 VG enriched with variants produces the major improvements in running time."*

We also modified accordingly **S9 Figure** caption as follows:

*"****S9 Fig. GRAFIMO running time efficiently scales with the number of threads used.*** *By running GRAFIMO with multiple threads (A) the running time significantly decreases, while (B) memory usage remains similar."*

3) You say that you have made all scripts for reproducing the experiments available in a dedicated Github repository, but in your comment you link to the GRAFIMO Github repository. Is this the wrong link? I cannot seem to find the shell scripts for reproducing the experiments in this repository.

We kindly thank the reviewer for raising that point. The scripts to reproduce the analysis are made available in a subdirectory of GRAFIMO Github repository.

We provide below the full link to scripts location:
https://github.com/pinellolab/GRAFIMO/tree/master/docs/paper_results

We have also added this note on the README on our main GitHub page:
"How to reproduce the experiments

All the scripts and IPython notebooks required to reproduce the experiments and the analysis presented in GRAFIMO's paper are available here."
