# Supplementary material for: GRAFIMO: Variant and haplotype aware motif scanning on pangenome graphs
Source: PLoS Comput Biol. 2021 Sep 27;17(9):e1009444. doi: 10.1371/journal.pcbi.1009444 (PMC8519448; doi:10.1371/journal.pcbi.1009444)
Supplement: Attachment — Submitted filename: Response_to_reviewers_grafimo.docx [file pcbi.1009444.s003.docx]

**Response to reviewers**

**Title:** “GRAFIMO: variant and haplotype aware motif scanning via pangenome graphs”

**ID:** PCOMPBIOL-D-21-00041

**Authors:** Manuel Tognon, Vincenzo Bonnici, Erik Garrison, Rosalba Giugno and Luca Pinello

We would like to thank the reviewers for the positive comments and feedback. We believe we have addressed all the points raised and the proposed revisions substantially improved the manuscript.

We provide below a point-by-point response and changes to the manuscript were highlighted in red.

**Reviewer 1**

**1) The authors presented a new tool to scan known TF DNA motifs in VG (graph genome). The manuscript overall is clear and the description is concise. The idea is not fully novel, for example similar study is reported here: (Cristian Groza, et. al. 2020 Genome Biology). Also, the tool itself heavily based on VG and overall is simple, but it do have many potential users.**

We kindly thank the reviewer for the comments and for considering our tool useful for a potentially large audience.

We have added the suggested reference and a brief sentence to the main text to report that the VG framework has been used before to model potential effects of genetic variants on regulatory regions captured by histone mark profiling:

*“Moreover, by encoding individual haplotypes, VGs have been shown to be an effective framework to capture the potential effects of personal genetic variants on functional genomic regions profiled by ChIP-seq of histone marks [12].”*

**2) The authors construct the VG from the 1000G data, which is a diverged dataset. Are there population specific TF motifs are found? What’s the ratio of that?**

We kindly thank the reviewer for suggesting this important analysis. Among the retrieved potential binding sites for the three considered TFs found only in individual haplotypes we observed a large fraction of population specific TFBS candidates by mapping the results back to the haplotypes from the 1000GP super populations. For CTCF several putative binding sites are specific for AFR (24.66%), EUR (6.74%), AMR (5.68%), SAS (13.01%) and EAS populations (12.52%). The remaining 37.38% are shared between two or more populations. Similarly, for ATF3 we observed that several potential binding sites are specific for AFR (17.07%), EUR (2.44%), AMR (9.76%), SAS (24.39%) and EAS (14.63%) populations. The remaining 31.71% of putative ATF3 binding sites are shared by two or more populations. Considering GATA1 we found that several potential TFBS are specific for AFR (25.97%), EUR (3.90%), AMR (9.09%), SAS (19.48%) and EAS (11.69%) populations. The remaining 29.87% of potential GATA1 TFBS are shared by two or more populations.

The manuscript was extended by adding this new text and adding a new subpanel (D) to **Fig 2**:

*“Interestingly, we observed that a large fraction of putative binding sites recovered only on individual haplotypes are population specific. For CTCF we found that 24.66%, 6.74%, 5.68%, 13.01%, 12.52% of potential CTCF TFBS retrieved on individual haplotype sequences only are specific for AFR, EUR, AMR, SAS and EAS populations, respectively (****Fig 2 (D)****). Similar results were observed for ATF3 and GATA1 (****S1 Text section 4-5****).”*

***Fig 2. Searching CTCF motif on VG with GRAFIMO provides an insight on how genetic variation affects putative binding sites.*** *(A) Potential CTCF occurrences statistically significant (P-value < 1e^-4^) and non-significant found in the reference and in the haplotype sequences found with GRAFIMO oh hg38 1000GP VG. (B) Statistical significance of retrieved* *potential CTCF motif occurrences and frequency of the corresponding haplotypes embedded in the VG. (C) Percentage of statistically significant CTCF potential binding sites found only in the reference genome or alternative haplotypes and with modulated binding scores based on 1000GP genetic variants (D) Percentage of population specific and common (shared by two or more populations) potential CTCF binding sites present on individual haplotypes.*

Similarly, we also added the following sentences to **S1 Text section 4 and 5** and added a subpanel (D) to **S5 and S6 Figs**:

*“We also observed that a considerable fraction of putative ATF3 binding sites are population specific. In fact, 17.07%, 2.44%, 9.76%, 24.39%, 14.63% of potential TFBS retrieved on individual haplotypes are specific for AFR, EUR, AMR, SAS and EAS populations, respectively (****S5 Fig (D)****).”*

***S5 Fig. Searching ATF3 motif on VG with GRAFIMO provides an insight on how genetic variation affects putative binding sites.*** *(A) Potential ATF3 occurrences statistically significant (P-value < 1e^-4^) and non-significant found in the reference and in the haplotype sequences found with GRAFIMO oh hg38 1000GP VG. (B) Statistical significance of retrieved* *potential ATF3 motif occurrences and their frequency in the haplotypes embedded in the VG. (C) Percentage of statistically significant ATF3 potential binding sites found only in the reference genome or alternative haplotypes and with modulated binding scores based on 1000GP genetic variants. (D) Percentage of population specific and common (shared by two or more populations) potential ATF3 binding sites present on individual haplotypes.*

*“We also found that several potential GATA1 binding sites among those retrieved only in individual genome sequences are population specific. We observed that 25.97% of putative binding sites are specific for AFR, 3.90% for EUR, 9.09% for AMR, 19.48% for SAS and 11.69% for EAS populations (****S6 Fig (D)****).”*

***S6 Fig. Searching GATA1 motif on VG with GRAFIMO provides an insight on how genetic variation affects putative binding sites.*** *(A) Potential GATA1 occurrences statistically significant (P-value < 1e^-4^) and non-significant found in the reference and in the haplotype sequences found with GRAFIMO oh hg38 1000GP VG. (B) Statistical significance of retrieved* *potential GATA1 motif occurrences and their frequency in the haplotypes embedded in the VG. (C). Percentage of statistically significant GATA1 potential binding sites found only in the reference genome or alternative haplotypes and with modulated binding scores based on 1000GP genetic variants. (D) Percentage of population specific and common (shared by two or more populations) potential GATA1 binding sites present on individual haplotypes.*

**3) For those discovered non-ref TF motifs, what’s the effect from different type/length of mutations? For example, do indel and larger mutations have larger effect?**

This is an excellent suggestion, and we performed the proposed analysis. For all the three TFs we didn’t observed a clear and general trend when comparing the effect of SNPs and indels of increasing length. We added a new figure in the supplementary material (**S7 Fig**) and updated the **Result** **section** in the main text with the following text:

*“We also investigated the potential effects of the different length and type of mutations i.e. SNPs and indels on the CTCF, ATF3 and GATA1 binding sites. However, we didn’t observe a clear and general trend (****S7 Fig****)****.”***

***S7 Fig. Influence of different type of mutations and length on binding affinity score.*** *(A) CTCF, (B) ATF3, (C) GATA1.*

**4) The authors need to benchmark the performance (memory/running time) of the tool.**

We kindly thank the reviewer for the suggestion. To systematically perform this analysis, we benchmarked the running time and memory usage of GRAFIMO in different scenarios and by comparing its performance to FIMO.

We searched the CTCF motif (19 bp, JASPAR ID MA0139.1) on 1000 regions of increasing length (1 to 9 millions of bp). Since FIMO does not provide a parallel implementation, we run GRAFIMO using one single thread. FIMO is faster and use less memory when searching the motif without accounting for genetic variants (to run GRAFIMO in such conditions we used a VG without encoded variants). However, when accounting for genetic variants belonging to the 2548 samples from 1000GP on GRCh38 phase 3, GRAFIMO proves to be faster than FIMO, since the latter would require 2548 individual runs of the tool to perform the same analysis (one for each sample). Moreover, we did not consider in the running time of FIMO the preprocessing steps required to retrieve the individuals’ genomic sequences based on the genetic variants stored in the VCF files. We also compared the differences in running time and memory usage using different numbers of threads, 1, 4, 8, and 16, respectively.

To report this new analysis, we added the following text in the main manuscript:

*“We designed GRAFIMO to efficiently search potential motif occurrences considering genetic variation present in panels of individuals.* ***S1 Text section 6*** *describes how the tool performance compares to those of a tool designed to work on a single genomic sequence at a time, such as FIMO [13], how it varies when using an increasing number of threads and its memory requirements (****S8 Fig*** *and* ***S9 Fig****).”*

Moreover, we added two new figures to the supplementary materials (**S8 Fig** and **S9 Fig**) showing the comparison between GRAFIMO and FIMO and the performance using different number of threads, respectively. We also extended the text in **Section 6** of the supplementary materials as follow:

“*We also benchmarked GRAFIMO comparing its running time and memory usage to FIMO. To this end we searched for potential occurrences of the CTCF motif (19 bp wide, JASPAR ID MA0139.1) on forward and reverse strand in 1000 genomic regions of the human chr22 of increasing length (1 to 9 million). To run FIMO, for each set of genomic regions we created FASTA file as required by this tool.*

*We assessed GRAFIMO performance scanning the chr22 VG without any encoded genetic variant for CTCF motif occurrences, on the previously computed sets of genomic regions. Moreover, since FIMO does not provide a parallel implementation, we run GRAFIMO using a single thread. In this scenario FIMO is faster and requires less memory than GRAFIMO (****S8 Fig (A)*** *and* ***(B)****).*

*We also benchmarked GRAFIMO running time with a single thread when performing a motif search on the VG encoded chr22 with 1000GP genetic variants (2548 individuals). In this test setting, excluding the preprocessing required to retrieve the FASTA genomic sequences for each of the 2548 1000GP subjects from the running time calculations, GRAFIMO proves to be faster, although FIMO speed is similar scanning regions up to 1 millions of bp (****S8 Fig (C)****).*

*These results are expected since FIMO is a highly optimized tool to scan linear genomic sequences, while GRAFIMO has been designed to efficiently scan panels of individuals simultaneously and to run in parallel using multiple threads.*

*We also assessed GRAFIMO running time and memory usage when using 1, 4, 8 and 16 threads, respectively. This analysis was performed using the chr22 VG with 1000GP genetic variants (****S9 Fig****).*

*As expected, running the tool with multiple threads resulted in a dramatical reduction of the running time at the cost of an increased usage of the memory. Not surprisingly, running GRAFIMO in parallel on the chr22 VG enriched with variants produces the major improvements in running time.*

*To obtain the results presented in the manuscript on average each scan required ~15 minutes and used ~24 GB of memory.”*

***S8 Fig. Comparing GRAFIMO and FIMO performance.*** *(A) FIMO is faster than GRAFIMO (using a single thread) when searching CTCF motif (JASPAR ID MA0139.1) on human chr22 regions (total width ranging from 1 to 9 millions of bp) and without accounting for genetic variants. (B) FIMO uses less memory resources than GRAFIMO, however it can only scan linear sequences. (C) GRAFIMO is generally faster than FIMO in searching potential CTCF occurrences after 1Mbp when considering genetic variation present in large panels of individuals as 1000GP on GRCh38 phase 3 (2548 samples), even with a single thread.*

***S9 Fig. GRAFIMO running time and memory usage.*** *(A) GRAFIMO running time significantly decreases when using multiple threads. (B) Memory usage increases when using multiple threads.*

**Reviewer 2**

**1) This is a well written manuscript, presenting a method that I think will be useful for many researchers in the field. GRAFIMO is a nice application of genome graphs, and the method provides a clear benefit compared to the existing "linear" method FIMO. I particularly like that you've designed GRAFIMO so that it can be used as a direct replacement to FIMO. I don't have many comments and I really don't have any major issues with the manuscript. However, I ran into some problems when trying to run the software and would like you to fix these so that I'm able to fully try out the software before concluding on the manuscript.**

We kindly thank the reviewer for the positive assessment of our tool, and we are very grateful for the extensive testing and for pointing out in detail the problems in running GRAFIMO. We believe we have fixed all the highlighted problems and create a new stable version ready to be tested and available at https://github.com/pinellolab/GRAFIMO.

**2) I have a few questions related to how you do false discovery rate adjustment of the p-values for each motif match. As far as I understand Fimo, q-values are found simply by accounting for the number of hypothesis tests done within the specified regions on the linear reference genome (i.e. one test for every base pair within the regions). I don't find any information in the manuscript or in the supplementary text on how you do this on a graph. I assume that you maybe account for all the hypothesis tests performed on the graph, i. e. the number of kmer-paths in the graph (following known haplotypes, if specified)? I know that FIMO has to hold all matches in memory in order to compute these p-values, and that the user can specify how many to hold in memory through the --max-stored-scores option. I don't see this option in GRAFIMO.**

We thank the reviewer for raising this point that required clarification. As the reviewer assumed, in GRAFIMO we explicitly account for all the hypothesis tests performed on the VG. Thus, while correcting *P-values* to *q*-values we keep in memory all the *k*-mers extracted within the paths (following the individual haplotypes encoded in the VG) corresponding to the genomic regions where we searched the motif. The *q*-values are computed using the Benjamini-Hochberg procedure. To clarify this point we added the following sentence to the manuscript under the **Transcription Factor motif search** subsection:

*“P-values are then converted to q-values by using the Benjamini-Hochberg procedure to account for multiple hypothesis testing. For this procedure, we consider all the P-values corresponding to all the k-mer-paths extracted within the scanned regions on the VG.”*

**3) Installation of Grafimo went fine on my system (used pip). I only had a minor issue with sphinx not being specified as a dependency in the setup.py file. Should that be added?**

We kindly thank the reviewer for pointing out this problem. As suggested, we included also sphinx among the required dependencies in the setup.py file.

**4) How does GRAFIMO compare to FIMO when it comes to run time? Is it considerably slower than FIMO, since it has to search for more potential motif matches? I personally don't mind if it is a bit slower than FIMO, but it would be nice if you could include a sentence about runtime. If runtime isn't an issue, that could be mentioned, and if runtime is an issue, it could be nice for the reader to know for instance how long time GRAFIMO and FIMO spends on processing e.g. one chromosome using one thread.**

We kindly thank the reviewer for the comment and suggestion to improve the manuscript. This is an important point raised also by Reviewer #1. Please see our reply to the point 4) above.

**5) If I only run "grafimo" on the command line, I get an IndexError (probably because I didn't specify -h or --help or anything else). It would be nice to instead get a more user friendly error, or just the help message.**

We would like to thank the reviewer for highlighting this issue with the tool. We changed the code and now by running “grafimo” without parameters a general help message is displayed.

**6) I tried running GRAFIMO on some of my own data, and I think I ran into some issues when running "grafimo buildvg" since it assumed I had chromosome chr1, chr2, chr3 and so on. My test dataset only had one chromosome "1" (not "chr1"). So I tried running "grafimo buildvg -h" to see if I could specify the chromosomes for my data. However, it seems that this doesn't give me the options for the subcommand "buildvg", but all the options for GRAFIMO? Or am I wrong? Anyway, it was a bit unclear for me how I could see which arguments only the buildvg command has.**

We kindly thank the reviewer for highlighting this problem. To fix this issue, we modified the GRAFIMO help message by reporting the general options (those who can be used with both the commands “buildvg” and “findmotif”), the “buildvg” options and the “findmotif” options. Moreover, we grouped together all the options available for a particular workflow.

**7) After specifying -c 1 to grafimo buildvg to try to make it only build graphs for chromosome 1, it seems that it tries to build a graph for "chr1". Would it maybe be better to let the user be able to specify the exact literal chromosomes, so that the user would need to specify "chr1" if chr1 is wanted (at least I often have data without the chr prefix). Right now it seems that chromosome "1" and so one (without chr) is not supported? EDIT: After I got another error (see next point), I tried installing the latest GRAFIMO through github, and it seems that things have changed there (from what is in the pip package)? On line 507 in constructVG.py, it seems that you remove "chr" from chromosome names, so when I now use "chr1" in my data, GRAFIMO crashes with "ValueError: Unknown chromosome given". Unless I have misunderstood something, it would be good if you fixed these issues. As a user, I would ideally be able to specify either "chr1" or "1", and GRAFIMO should then support both (and not convert the chromosome names).**

We kindly thank the reviewer for pointing out this issue. We fixed the problem described above and introduce the possibility to name in different ways the VG chromosomes.

Before building the VG, it is important that chromosome names style on the reference genome FASTA and VCF files match (e.g. if chromosome 1 is called >1 in FASTA reference, it should be named 1 in the VCF).

Moreover, we replaced the old flags -c and --chroms with new options specifically related to the selected workflow (*buildvg* or *findmotif*):

- --chroms-build chromosomes for which the VG will be built (names as in input FASTA reference).
- --chroms-prefix-build prefix to append at the front of chromosome names to identify the corresponding VGs. For example by using --chroms-prefix-build “”, the corresponding VGs files will be called 1.xg, 2.xg, etc.
- --chroms-namemap-build defines a space- or tab-separated file containing chromosome name in the first column and the names to use to store the corresponding VGs in the second column.
- --chroms-find chromosome to consider during motif search.
- --chroms-prefix-find prefix shared by all chromosome VGs.
- --chroms-namemap-find defines a space- or tab-separated file containing chromosome name in the first column and the names used to identify the corresponding chromosome VGs.

**8) After solving the issues with chromosome by using other data (now data with "chr1"), I got the following error message when using the example.meme file:
File "/usr/local/lib/python3.8/dist-packages/grafimo/motif.py", line 634, in read_MEME_motif
motifID, motifName = line.split()[1:3]
ValueError: not enough values to unpack (expected 2, got 1)
When checking line 634 in motif.py in your github-repo, I realised that this might be an old bug that is fixed since the pip-package was published. That's when I decided to pull your latest code from Github, and I then ran into issues with "chr1" not being supported anymore. At this point I gave up, but I guess there's not much that needs to be fixed on your side before I should be able to run GRAFIMO. Let me know if you believe I've misunderstood anything or done anything wrong.**

We kindly thank the reviewer for highlighting this potential issue and for the patience in going through these problems. We were able to reproduce this error, this was due the assumption that a MEME file should always contain both the motif ID and the motif alternative name. We have fixed this problem and GRAFIMO now also supports MEME files having only the motif ID.

**9) I think it is very nice that you have included so many details about the experiments in the supplementary text, but I don't find any scripts for reproducing the experiments. It would be nice with reference to scripts (and ideally also the graphs) you used for running the experiments.**

We kindly thank the reviewer for the comment. We believe reproducible research is very important therefore we made available on a dedicated GitHub repository: <https://github.com/pinellolab/GRAFIMO>:

1) Shell scripts to download the VGs we used in our experiments and used in all the analysis presented in the manuscript.
2) Shell scripts to reproduce the analysis presented in the manuscript.
3) A Jupyter Notebook to showcase the results presented in the paper.

In addition, we extend the section *Availability and Future Directions* as follow:

*“The genome variation graphs enriched with 1000GP on GRCh38 phase 3 used to obtain all the results presented in this manuscript can be downloaded at* [*http://ncrnadb.scienze.univr.it/vgs*](http://ncrnadb.scienze.univr.it/vgs)*.”*
